# Supplementary material for: Association of Body Composition with Tumor Proteomics and Survival in Patients with Clear Cell Renal Cell Carcinoma
Source: Cancers (Basel). 2026 Sep 30;18(19):3161. doi: 10.3390/cancers18193161 (PMC13664272; doi:10.3390/cancers18193161)

Supplementary Table S1: Descriptive statistics comparison between included patients and excluded patients.

Characteristic	Total	Included in Analysis	Excluded	p-value[2]
	N = 537[1]	N = 199[1]	N = 338[1]	
Age At Diagnosis (Years)	61 (52, 70)	61 (51, 71)	61 (52, 70)	0.6
(Unknown/Missing)	35	15	20	
Sex				0.8
Female	191 (36%)	69 (35%)	122 (36%)	
Male	346 (64%)	130 (65%)	216 (64%)	
Race				0.2
Asian	8 (1.5%)	2 (1.0%)	6 (1.8%)	
Black Or African American	56 (11%)	15 (7.5%)	41 (12%)	
White	466 (88%)	182 (91%)	284 (86%)	
(Unknown/Missing)	7	0	7	
Ethnicity				0.5
Hispanic Or Latino	26 (6.8%)	11 (7.9%)	15 (6.1%)	
Not Hispanic Or Latino	359 (93%)	129 (92%)	230 (94%)	
(Unknown/Missing)	152	59	93	
AJCC Pathologic Stage				0.13
Stage I	235 (53%)	86 (53%)	149 (52%)	
Stage II	53 (12%)	12 (7.5%)	41 (14%)	
Stage III	103 (23%)	43 (27%)	60 (21%)	
Stage IV	56 (13%)	20 (12%)	36 (13%)	
(Unknown/Missing)	90	38	52	
Primary Diagnosis				0.7
Adenocarcinoma, Nos	6 (1.2%)	1 (0.6%)	5 (1.6%)	
Adenosquamous Carcinoma	1 (0.2%)	1 (0.6%)	0 (0%)	

Carcinoid Tumor, NOS	1 (0.2%)	0 (0%)	1 (0.3%)	
Chronic Lymphocytic Leukemia	1 (0.2%)	0 (0%)	1 (0.3%)	
Clear Cell Adenocarcinoma, NOS	446 (92%)	158 (92%)	288 (92%)	
Clear Cell Carcinoma	3 (0.6%)	1 (0.6%)	2 (0.6%)	
Malignant Lymphoma, Non-Hodgkin, NOS	1 (0.2%)	1 (0.6%)	0 (0%)	
Malignant Melanoma, NOS	1 (0.2%)	1 (0.6%)	0 (0%)	
Melanoma, Nos	1 (0.2%)	0 (0%)	1 (0.3%)	
Mucinous Adenocarcinoma	1 (0.2%)	1 (0.6%)	0 (0%)	
Neuroendocrine Carcinoma, NOS	1 (0.2%)	1 (0.6%)	0 (0%)	
Papillary Carcinoma, Follicular Variant	1 (0.2%)	1 (0.6%)	0 (0%)	
Papillary Microcarcinoma	1 (0.2%)	0 (0%)	1 (0.3%)	
Pituitary Adenoma, NOS	1 (0.2%)	0 (0%)	1 (0.3%)	
Renal Cell Carcinoma, NOS	13 (2.7%)	4 (2.3%)	9 (2.9%)	
Squamous Cell Carcinoma, NOS	3 (0.6%)	1 (0.6%)	2 (0.6%)	
Squamous Cell Carcinoma, Clear Cell Type	1 (0.2%)	0 (0%)	1 (0.3%)	
Transitional Cell Carcinoma	1 (0.2%)	0 (0%)	1 (0.3%)	
Transitional Cell Carcinoma in Situ	1 (0.2%)	0 (0%)	1 (0.3%)	
(Unknown/Missing)	52	28	24	
Vital Status				0.4
Alive	360 (67%)	138 (69%)	222 (66%)	
Dead	177 (33%)	61 (31%)	116 (34%)	
[1] Median (Q1, Q3); N (%)				
[2] Wilcoxon Rank Sum Test; Fisher's Exact Test				
NOS: Not Otherwise Specified				

Supplementary Table S2: Tissue radiodensity cutoff points in Hounsfield units for computed tomography image annotation

Tissue	Tagged tissue locations	Lower limit of Hounsfield unit	Upper limit of Hounsfield unit
Subcutaneous adipose tissue	Subcutaneous fat	-190	-30
Intermuscular Adipose tissue	Intermuscular fat	-190	-30
Visceral adipose tissue	Fat around organs	-150	-50
Total skeletal muscle	Skeletal muscle	-29	200
Bone	Bone	200	2001

Supplementary Table S3: Risk of mortality for covariates

Variable	HR (95% CI)	P-Value
Sex (Reference: Female)	2.28 (1.16-4.45)	0.016[1]
Age: Step Size: 10 years	1.32 (1.03-1.70)	0.027[2]
Race (Reference: White)		0.714[1]
Asian	1.23 (0.16-9.55)	0.842[2]
Black or African American	0.44 (0.06-3.34)	0.424[2]
Ethnicity (Reference: Hispanic or Latino)		<.001[1]
Not Hispanic or Latino	5.20 (0.69-39.31)	0.110[2]
Not Reported	16.31 (2.04-130.17)	0.008[2]
AJCC Pathologic Stage (Reference: Stage I)		<.001[1]
II	0.93 (0.26-3.35)	0.912[2]
III	3.77 (1.85-7.71)	<.001[2]
IV	8.46 (4.12-17.37)	<.001[2]

[1]Type 3 Wald p-value; [2]Covariate Wald p-value.
All models were adjusted for all other covariates and body composition groups.
Abbreviations: AJCC, American Joint Committee on Cancer; CI, confidence interval; HR, hazard ratio.

Supplementary Figure S1: Samples of Excluded images.

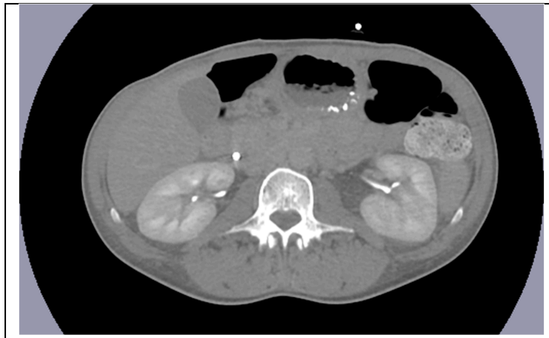

Poor resolution image (raw)

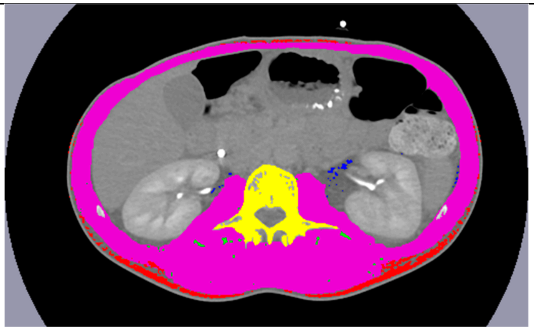

Poor resolution image (segmented – VAT out of HU range)

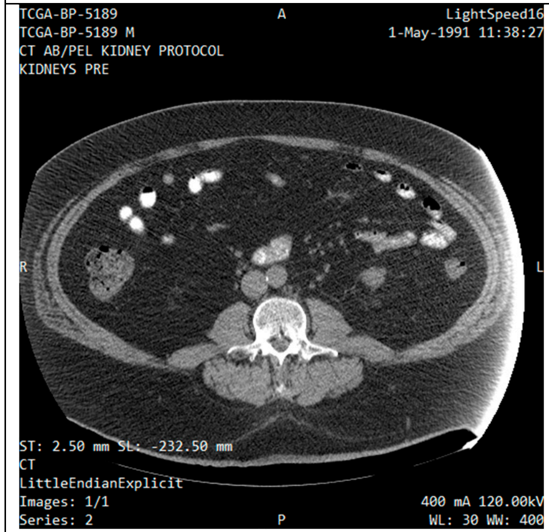

Truncated image with right peripheral edge hyperradiodensity (raw)

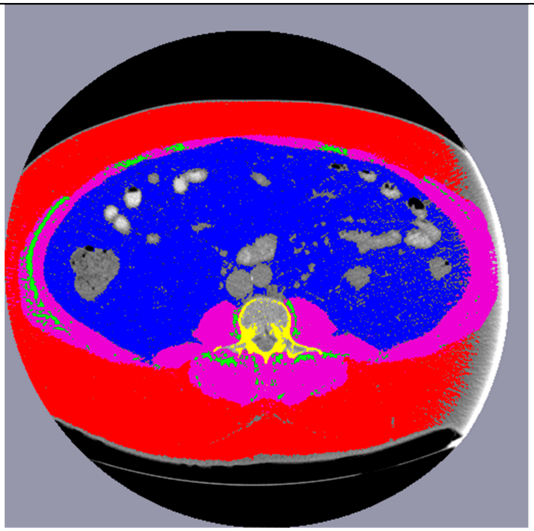

Truncated image with right peripheral edge hyperradiodensity (segmented)

Supplementary Figure S2: Flowchart of patient selection from the TCGA-KIRC cohort

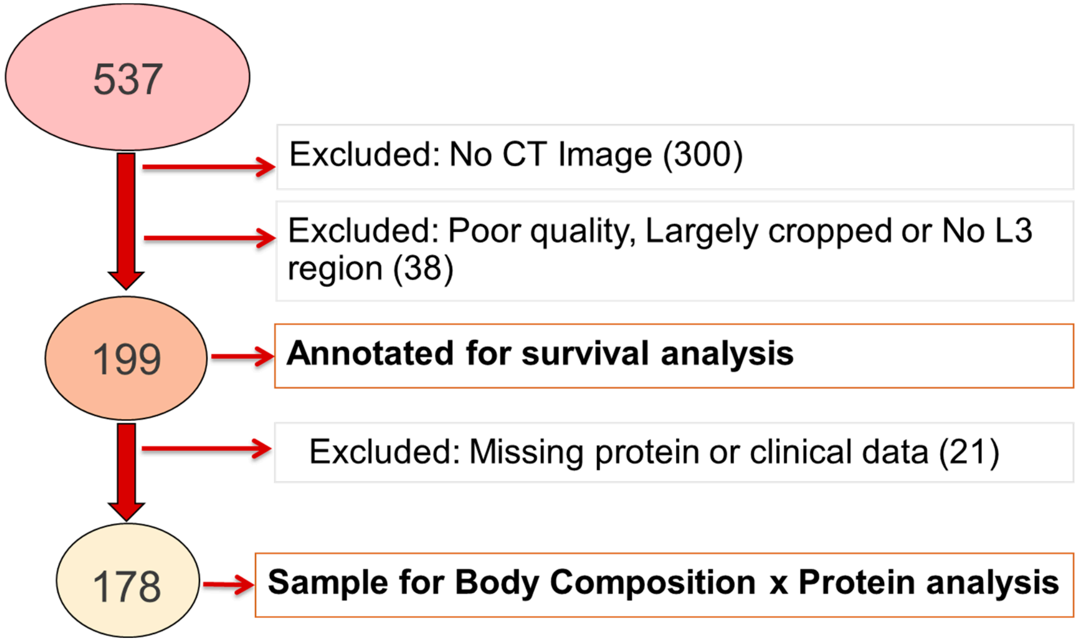

Abbreviations: CT, computer tomography; L3, third lumbar vertebra

Supplementary Figure S3: Evaluation of the Proportional Hazards Assumption Across Body Composition Categories

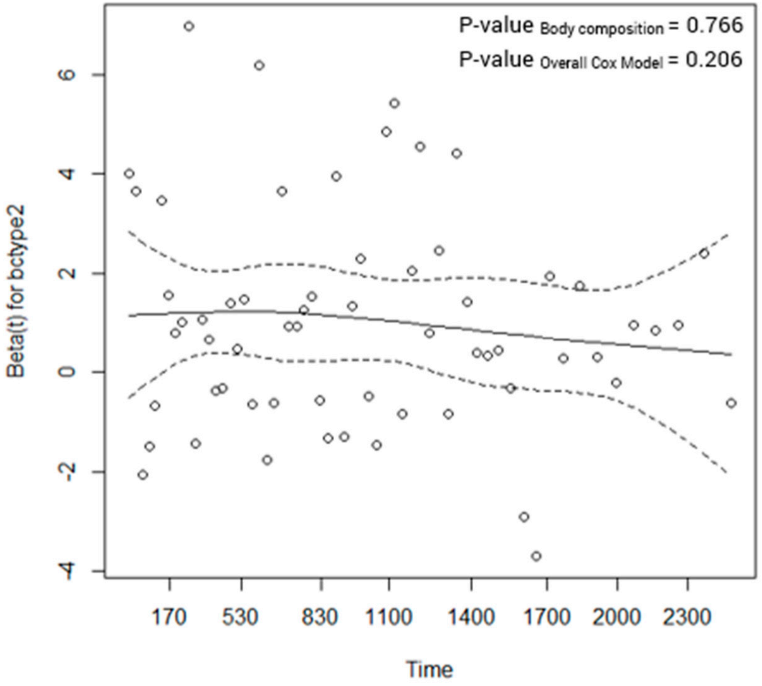

Supplementary Figure S4: Total muscle area cutoffs

A. FEMALE

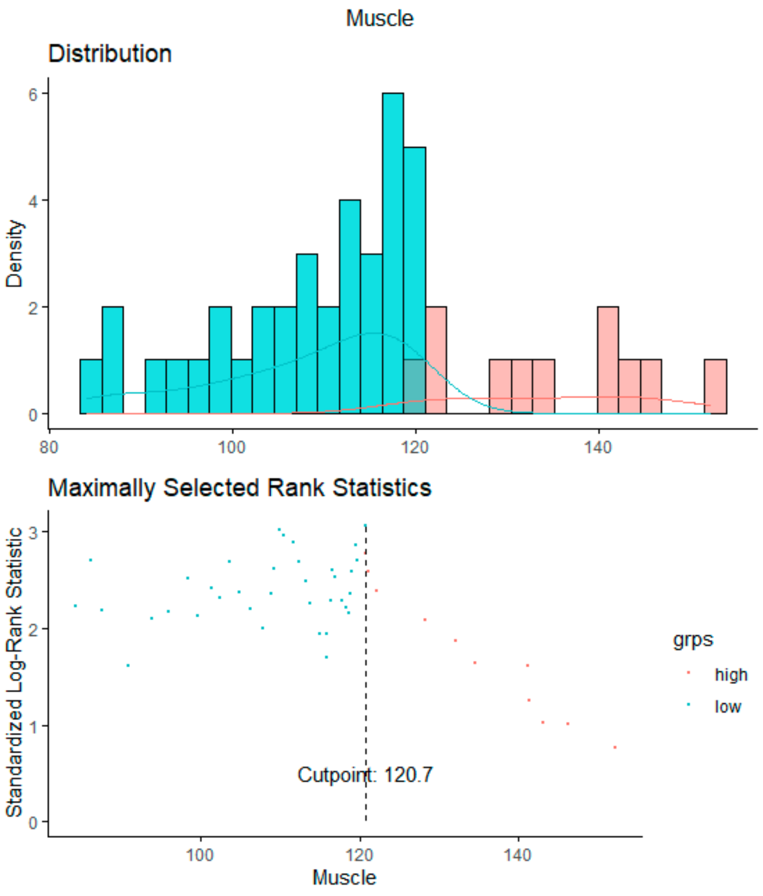

B. MALE

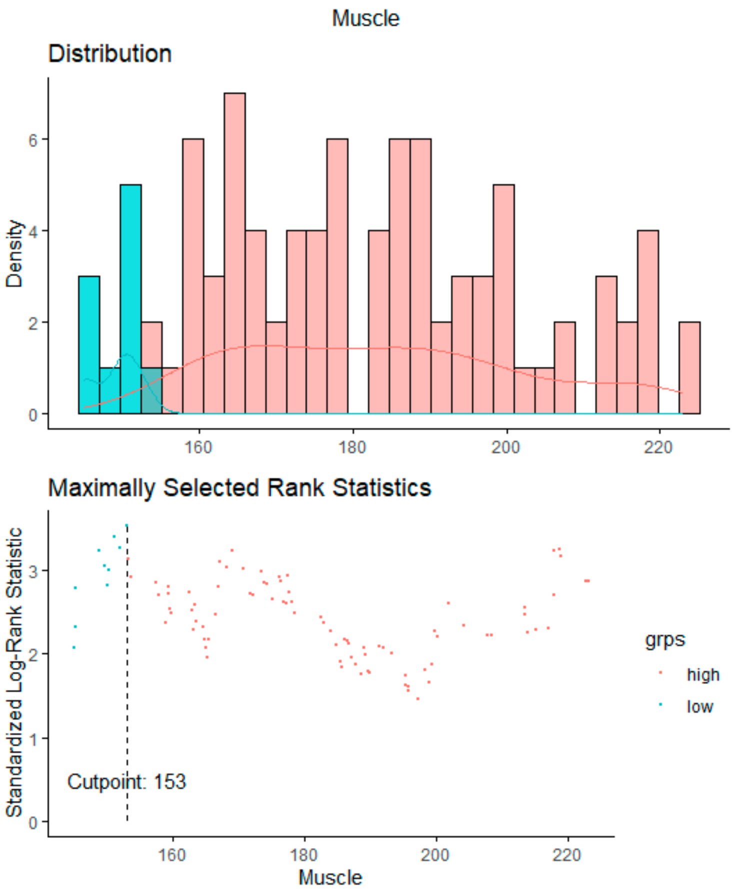

Supplementary Figure S5: Total adipose tissue cutoffs

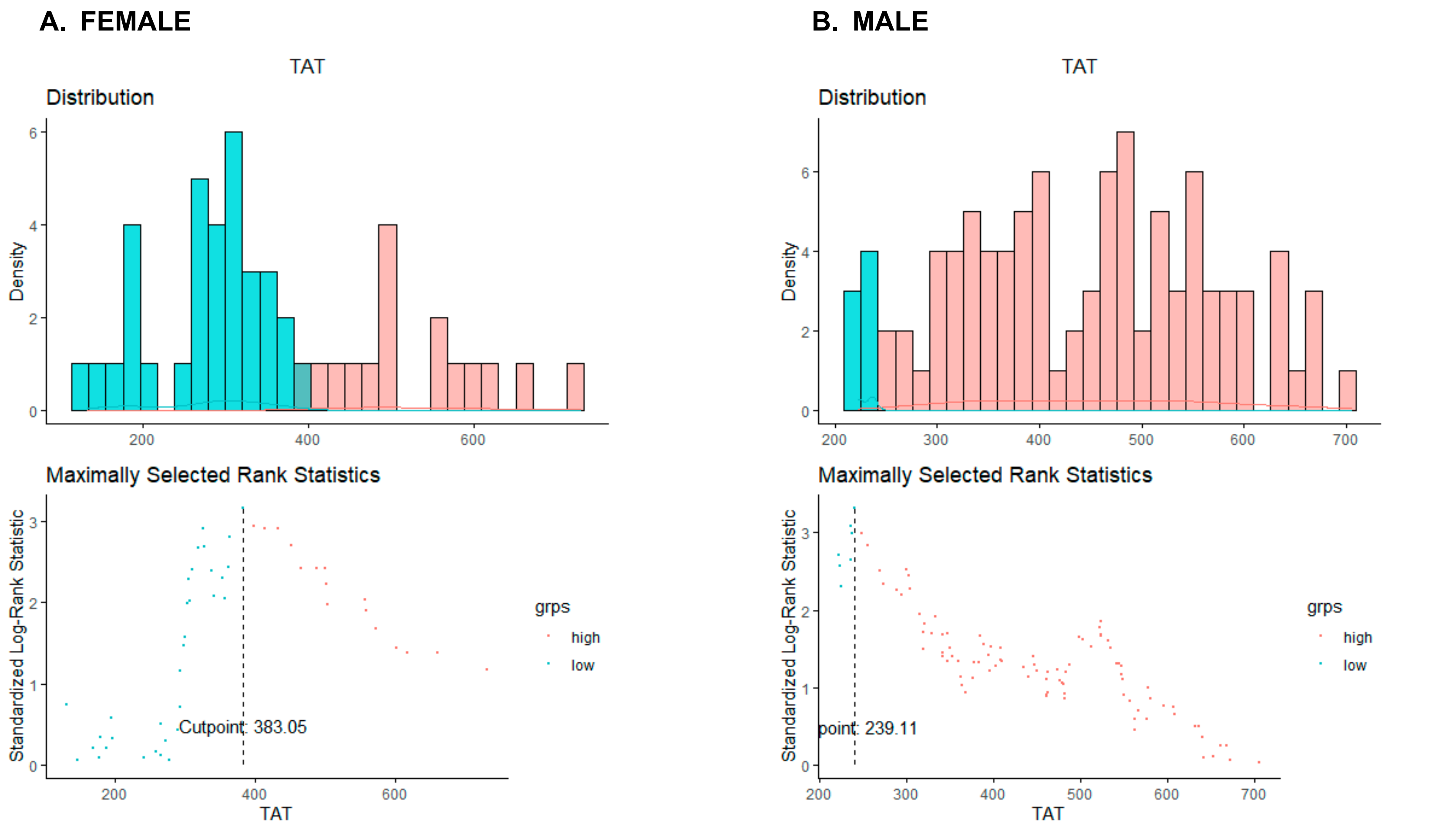

Supplementary Figure S6: PREX1 distribution and corresponding Kaplan-Meier survival curve

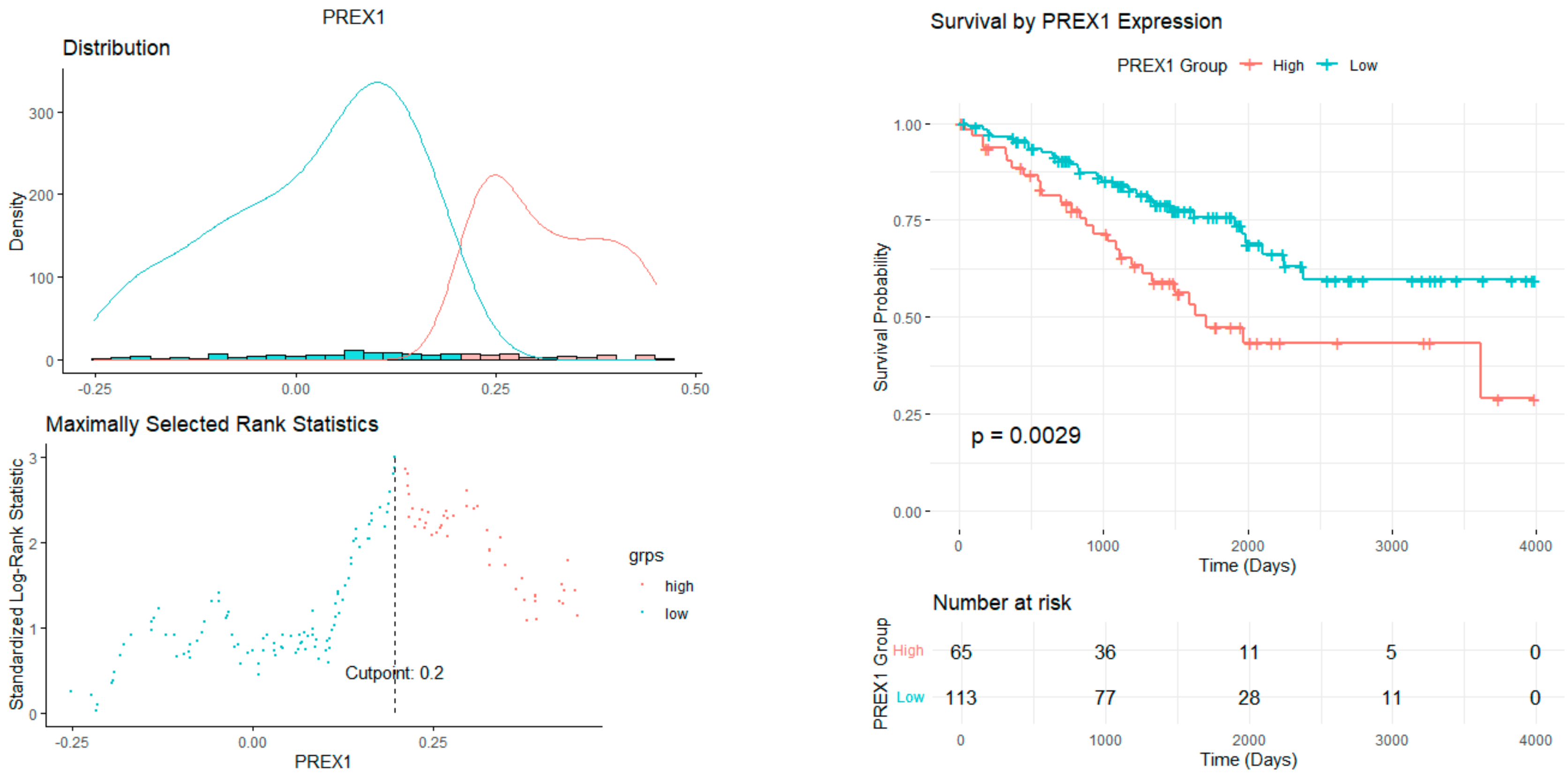

Supplement: Supplementary file 1 [file cancers-18-03161-s001.zip › Supplementary Tables and figures.pdf]
